# Recent Progress in Biomedical Sensors Based on Conducting Polymer Hydrogels

**DOI:** 10.1021/acsabm.3c00139

**Published:** 2023-04-28

**Authors:** Jillian Gamboa, Sofia Paulo-Mirasol, Francesc Estrany, Juan Torras

**Affiliations:** Departament d’Enginyeria Química, EEBE, Universitat Politècnica de Catalunya, C/Eduard Maristany, 10-14, Ed. I.2, Barcelona 08019, Spain

**Keywords:** biomedical, hydrogel, conducting polymer, biosensor, wearable, implantable

## Abstract

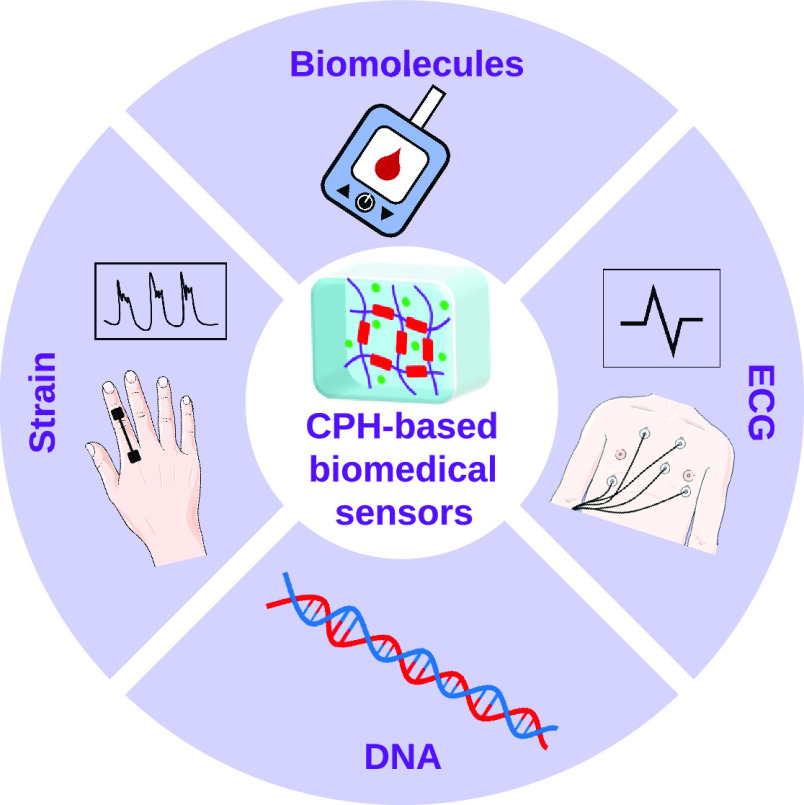

Biosensors are increasingly taking a more active role in health science. The current needs for the constant monitoring of biomedical signals, as well as the growing spending on public health, make it necessary to search for materials with a combination of properties such as biocompatibility, electroactivity, resorption, and high selectivity to certain bioanalytes. Conducting polymer hydrogels seem to be a very promising materials, since they present many of the necessary properties to be used as biosensors. Furthermore, their properties can be shaped and enhanced by designing conductive polymer hydrogel-based composites with more specific functionalities depending on the end application. This work will review the recent state of the art of different biological hydrogels for biosensor applications, discuss the properties of the different components alone and in combination, and reveal their high potential as candidate materials in the fabrication of all-organic diagnostic, wearable, and implantable sensor devices.

## 1 Introduction

The research and interest in the biosensing field has been growing quite rapidly in the recent years, with integration and miniaturization as two of the pillars of the new research, together with the development of novel materials.^1,2^ Thus, modern biosensors are combining the interesting properties of nanomaterials, such as metal or semiconductor nanoparticles (NPs), with biomaterials that exhibit unique recognition functions to assemble specialized bioanalytical and advanced bioelectronic devices.^3^

On the other hand, healthcare expenditures are continuously increasing due to population expansion from increased life expectancy, resulting in higher costs of services, especially inpatient hospital care. Hence, novel biosensors could help reduce these costs by favoring preventive healthcare.^4^ Indeed, the total world healthcare expenditure in 2019 represented around 9.8% of the global gross domestic product (GDP), having doubled over the last two decades. However, this value was unequally distributed, as higher income countries accounted for approximately 80% of the expenditure.^5^ It is worth noting that the growth in health care spending seen in recent times emphasizes the need for new and more reliable low-cost and easy-to-use diagnostic tools. Taking a closer look at socioeconomic data, the global market size for the development of novel biosensors was about $26.9 billion by 2022 and is expected to reach $49.8 billion by 2030. Novel biosensors are thus recognized as invaluable tools in the new health industry.^6^ An example of this trend could be the extended use of glucose biosensors, which represent 85% of the total biosensor market. In recent years, glucose biosensors have significantly improved the quality of life of diabetic patients, with a very important socioeconomic impact.^7^ Similarly, wearable technologies based on implantable solutions are increasingly adopted, which can provide real-time information and new therapeutic strategies on demand at the point of care. These new technologies clearly help to reduce healthcare costs.^6,8^ Therefore, an acceleration in the use of on-body and/or in vivo technologies is expected in the near future.^9^ Society demands progress toward a new paradigm of treatment that is less reliant on endoscopy and surgery and more focused on implantable sensors to help make diagnoses and guide drug administration. Implantable sensors are used to monitor biochemical and biophysical conditions inside a body, especially for patients with severe diseases. Depending on their purpose, the sensors are kept in the body from a matter of months to years or even for the patient’s entire lifetime in some cases. However, most traditional implanted sensors require a secondary surgery for retrieval after serving their purpose. This secondary surgery not only increases surgery risks but also increases hospitalization costs; hence, the need for bioresorbable sensors arises. Bioresorbable sensors are designed to degrade or resorb once they complete their intended function after a specific amount of time in the body.^10^

The type of bioresorbable material to be used in biosensors will depend mainly on the composition and function of each of their parts. Biosensors are composed of two main parts: the biorecognition element and the transducer element.^11^ The biorecognition element, which detects the biochemical or biophysical signal, is made of macromolecules such as proteins and enzymes embedded in polymers, while the transducer element or electrode, which transforms the physiological or biological signal into an electrical signal, can be made of metals, inorganic semiconductors, and polymers. Among the new materials that have gained considerable interest in the biosensing field are the group of materials known as conducting polymer hydrogels (CPHs). These polymeric materials synergistically combine the properties of conductive polymers and those of hydrogels. CPHs exhibit unique electrical, chemical, and mechanical properties, which give them great potential for use in the fabrication of different types of sensors. For instance, they can undergo physical and/or chemical transitions when exposed to changes in temperature, pressure, light, humidity, and pH, as well as in the presence of certain chemical/biological compounds.^12^ Furthermore, CPHs based on natural biopolymers combine the biocompatibility and biodegradability of biopolymers with the electroactive properties of conductive hydrogels, resulting in the applicability of CPHs for the fabrication of biodegradable and biocompatible sensors.^12^

**Figure 1:**
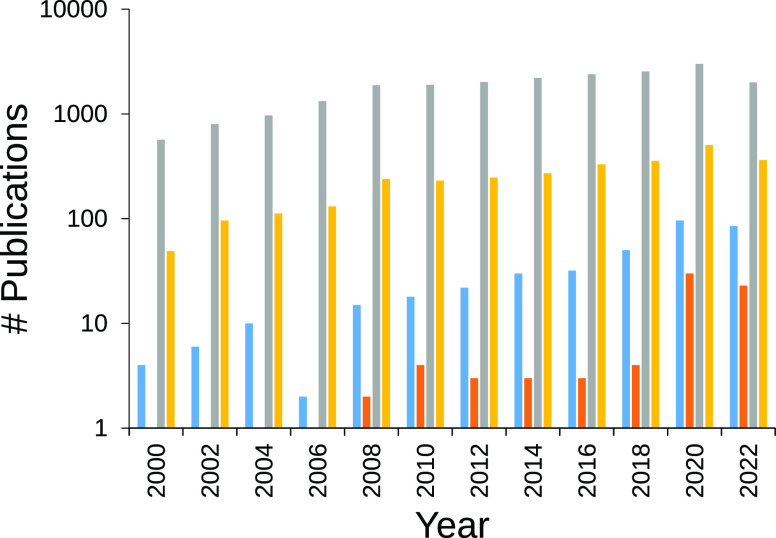
Number of publications (semi-logarithmic axes) in scientific databases obtained after searching for “conducting polymer hydrogel” (in blue), “conducting polymer hydrogel sensor” (in orange), “conducting polymer” (in gray), and “conducting polymer sensor” (in yellow) keywords.

The interest aroused by this type of material is evident in the evolution of scientific studies carried out with CPHs, which has only grown in the past decade. A close inspection of the literature by means of specific keywords to differentiate conducting polymers (CPs) from CPHs and their application to sensors shows a very interesting growth in publication trend (Figure 1). Moreover, the most specific scientific studies aimed at the use of CPHs as sensors have had a very significant increase in the last two or three years, where the number of papers has multiplied by six compared to those published in the 2018–2019 biennium. This sudden change in trend demonstrates the importance and interest that this type of material has been receiving for its more specialized use in sensors.

One of the main applications of CPHs in biomedicine, which is receiving greater recognition and impetus in recent years, would be their main use as biosensors. Indeed, CPHs in biomedical sensors can be found independently, within a portable and autonomous device, or as implantable sensors. The new trends are very ambitious, not only for the current recognition of bioanalytes such as dopamine (DA), glucose,^13^ DNA,^14^ and tumor markers,^15^ among others, but also due to the growing social demand for noninvasive, real-time measurements.^16^ The current healthcare trend is moving toward the real-time collection of patient-specific data to deliver the right medical treatment in a timely manner. Wearable devices are widely used within healthcare paradigms, with CPHs playing important roles in the new sensors industry due to their flexibility, biocompatibility, and high biodegradability potential (the FBB rule). For example, CPHs can be integrated into wearable pressure sensors,^17^ underwater strain sensors,^18^ finger movement detection,^19^ and novel sensitive photothermal hydrogels that transform visible radiation into heat.^20^ In addition, another important pool of applications with a promising future due to the interesting capabilities offered by the FBB rule is implantable sensors. Nowadays, sensors introduced in the human body are being developed using new and nontraditional materials such as fully organic probes,^21^ CPH electrodes,^22^ 3D-printable CPH ink,^23^ and biodegradable electronic circuits printed by photolithography.^24,25^

In this Review, we discuss the recent development of conducting polymer hydrogel-based biosensors and their applications. First, the components of the CPHs are described in detail, i.e., the conducting polymers, hydrogels, and their most recent applications in the field, thus showing their main properties to be used in sensor development. To this end, different types of CPs and hydrogels, both natural and synthetic, are described, most of which are used in sensing. Furthermore, main advances in the development of detection devices using different types of CPHs are visited. Finally, the most recent progress using CPHs in the development of isolated sensors, embedded sensors in wearable devices, and implantable sensors is shown.

## 2 Conducting Polymers

**Figure 2:**
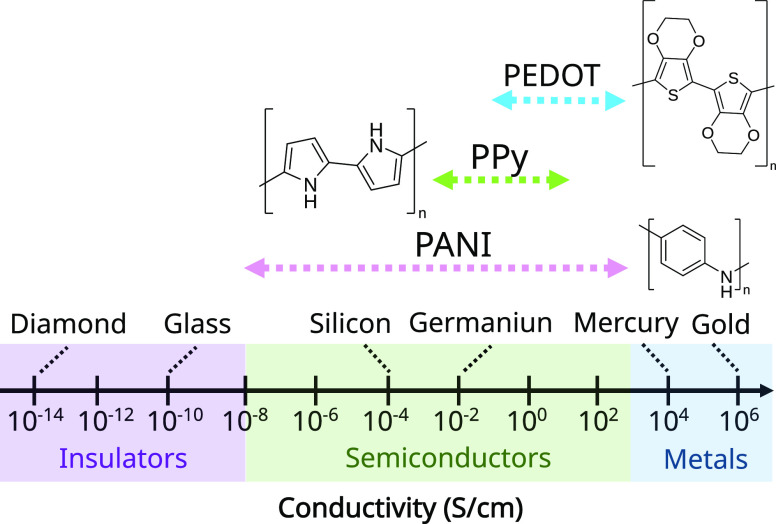
Conductivity ranges and structures of the three most commonly used CPs. The figure is adapted from ref (33). Copyright 2015 Royal Society of Chemistry. The figure is also adapted from ref (34). Copyright 2019 AIP Publishing.

Ever since the discovery of polyacetylene in 1977, the view of polymers as plastic insulators fundamentally changed, and the research on CPs has since been on the rise. CPs represent a unique class of polymers whose electrical and optical properties are similar to those of semiconductors.^26^ Most CP backbone chains consist of alternating single and double bonds made up of either polyenes or aromatic rings, which give their backbones a conjugated structure (Figure 2).^27^ In their pristine state, CPs present very low conductivity, which can increase multifold upon doping. Unlike their inorganic counterparts, doping of CPs results from redox reactions. CP chains with conjugated double bonds are capable of hybridizing at the nanoscale with carbon nanotubes (CNTs), graphene, metal NPs, or metal oxide NPs to achieve species with better adsorbent, catalytic, and interaction properties with other compounds, producing specific high-efficiency sensors.^28^ With doping, the conjugation of the double bonds of the polymer chains is stabilized and an excess or defect of electrons is introduced in the main chain. This allows the π-electrons of the double bonds of the CP structure to get displaced and move along the polymer chain, resulting in a flow of electrons. This gives CPs their conducting ability. CPs can be p-doped or n-doped. In p-doping, oxidation takes place.^29^ Electrons move from the highest occupied molecular orbital (HOMO) of the polymer to the dopant, resulting in a loss in electrons. On the other hand, n-doping results in electrons from the dopant migrating to the lowest unoccupied molecular orbital (LUMO) of the polymer. This then leads to an increase in electron density, thereby enabling electron conduction.^27^ Because of this, the conductivity of a CP relies not only on the arrangement and length of the polymer chain but also on the type and concentration of the dopant and the doping time.^30−32^ Aside from the addition of chemical dopants, doping of CPs can also be performed via electrochemical, photo, nonredox, and charge-injection methods.^27^ Examples of commonly studied CPs include polyaniline (PANI), polypyrrole (PPy), and poly(3,4-ethylenedioxythiophene) (PEDOT).

### 2.1 Polyaniline

Polyaniline has been a widely studied conducting polymer since the discovery of its conductive properties in the 1980s.^35^ It offers several advantages including high thermal and environmental stability, high conductivity, and simple and inexpensive synthesis routes.^36^ PANI can be synthesized through electropolymerization or chemical polymerization in the presence of a dopant, typically ammonium persulfate (APS).^37^ PANI can be found in four different states—leucoemeraldine base, pernigranaline base, emeraldine base, and emeraldine salt—depending on its oxidation and doping state. The first two states are the insulating states, while the last two are conducting states based on doping and dedoping of the chain due to the acidic and basic substances in the environment.^38^ Because of this property, PANI has been extensively used in pH sensors.^39^ For example, in a recent study, PANI was electrodeposited on a carbon micromesh electrode (CME) as the pH-sensitive component of a wearable, transparent, gas-permeable ascorbic acid and pH sensor. The resulting sensor demonstrated good sensing capabilities in a wide range of ascorbic acid concentrations (1 μM to 1 mM) with a low detection limit of 10 nM, a storage stability of at least 30 days, and a pH sensitivity and stability of 58.1 mV/pH and 0.33 mV/pH, respectively, when used in artificial tears. In addition, the group also integrated a reference electrode through the electrodeposition of silver nanoparticles onto the CME. The resulting linear equations from both ascorbic acid and pH sensing using the integrated reference electrode were similar to those obtained using a commercial silver–silver chloride (Ag|AgCl) reference electrode. Aside from pH sensing, PANI has also been employed in the fabrication of sensors for hazardous compounds such as volatile organic compounds^40^ and methanol vapors^41^ and in biomedical sensors for the detection of biomolecules such as creatinine^42^ and various cancer markers.^43^ For instance, Vural et al.^44^ modified a graphite electrode with a composite of PANI, a gold NP, and a peptide nanotube to immobilize an antiprostate-specific antigen for the detection of prostate specific antigen. This amperometric-based immunosensor could detect prostate-specific antigen in blood samples, with a maximum relative error of 14% compared to a commercially available enzyme-linked immunosorbent assay (ELISA) technique. In a similar study, a thin film of PANI was used as the conductive matrix for the tumor necrosis factor-α (TNF-α) antibody (Ab) as a point-of-care sensor for the detection of early osteoarthritis joint inflammation.^45^ Quantification of TNF-α could be performed due to changes in the electrochemical impedance spectroscopy (EIS) signals from the adsorption of TNF-α onto the Ab-PANI matrix. The resulting sensor exhibited excellent selectivity toward TNF-α in the presence of nonspecific analytes when tested with mouse serum, with an ±8% deviation from ELISA results. In another study, PANI was doped with nitrogen-doped graphene quantum dot (N-GQD) to fabricate a wearable noninvasive sweat glucose sensor.^46^ This combination provided a flexible matrix that allowed good adhesion of glucose oxidase (GOx) thanks to PANI, with improved electrical conductivity thanks to the N-GQD doping. The resulting sensor exhibited a sensitivity of up to 68.1 ± 1.11 μA mM^–1^ cm^–2^ for glucose in artificial sweat. In addition, it retained 92% of its sensitivity during simulated body movements, 89.4% after a 30 day storage period, and also showed good selectivity toward glucose in the presence of other bioanalytes commonly found in sweat such as uric acid (UA), ascorbic acid, and lactate. A recent study by Ouyang et al. also showed the possible use of PANI in wearable sensors.^47^ In their work, smart textiles were fabricated from silk yarns functionalized by PANI and carbon nanotubes (CNT), which endowed the silk yarns with conductive properties and sensitivity to temperature, motion, and environmental gases. One of the interesting findings in this study was that the internal structures of the silk yarns were well maintained after the functionalization. In addition, their functionality and structure integrity were also maintained after being sewn into textiles and exposed to water, friction, and deformation. The resulting smart textile could detect human motions when attached to various body parts and even showed changes in resistivity during different movement angles and velocity. As a temperature sensor, the smart textile could sensitivity of up to 4.05% °C^1–^, while as a gas sensor it exhibited sensitivity toward some volatile organic compounds, including ammonia and toluene.

### 2.2 Polypyrrole

Polypyrrole is another well-studied CP with a conductivity of up to 300 S cm^–1^, depending on the dopant used and its form.^48^ PPy has been synthesized in various forms, including both thick and thin films and nanosized tubes, particles, and fibers.^49−52^ Polymerization of PPy can be induced through electrochemical initiation by an anodic current, photoinduction, and chemical activation by oxidative reagents.^53−55^ Apart from its simple and inexpensive synthesis methods, PPy offers other advantages including biocompatibility, nontoxicity, commercial availability of reactants, and good environmental stability. These properties, together with its excellent electrochemical response, make PPy an excellent base polymer for the preparation of high-efficiency supercapacitors,^56^ semiconductors, and electrochemical sensors.^57,58^ However, PPy suffers from poor elasticity, tensile strength, and processability; hence, it is usually combined with other polymers or materials that can improve its mechanical properties, making it more suitable for a number of interesting biomedical applications.^59^ For instance, PPy was polymerized on the surface of a leather band to fabricate a wearable electrocardiogram (ECG) electrode.^60^ In this work, the authors showed that not only did PPy impart conductivity onto leather but it also improved its intrinsic antimicrobial properties. Moreover, this PPy–leather electrode could detect weak signals, comparable to commercially available electrodes, and was found to be more stable compared to commercially available gel-assisted electrodes. In another study, PPy was polymerized in the presence of polydopamine (PDA) on the surface of gold electrodes.^61^ In this work, the authors showed that the combination of the two materials resulted in a good biocompatibility and conductivity trade-off such that the growth and differentiation of myoblasts and neuronal cells improved in the presence of this combination. Furthermore, in vivo tests in mice showed good electromyogram (EMG) signals, demonstrating the possibility of using the material as a tissue engineering scaffold and as an implantable electrode. In another study, an indium titanium oxide (ITO) surface was modified with PPy, gold nanoparticles, and antibodies to fabricate an electrochemical sensor for systemic sclerosis (SSc).^62^ The resulting nanoimmunosensor could differentiate between the SSc-related biomarkers from different sets of patients with a limit of detection (LOD) of 0.42 pg mL^–1^, which is better than that of the gold standard, ELISA, and gave reproducible results with less than 4% of deviation. Similarly, the group of Aydin et al.^201^ also fabricated an immunsensor based on reduced graphene oxide/amino-substituted PPy for the detection of calreticulin (CALR), a novel biomarker for breast cancer, via EIS measurements. The resulting sensor exhibited a wide detection range from 0.025 to 75 pg mL^–1^, good selectivity in the presence of interferences, a good storage stability, and a LOD of 10.4 fg mL^–1^. Moreover, due to its nonspecific nature, this sensor fabrication method could possibly be employed for other diseases. Aside from these, PPy has also been used in the fabrication of sensors for bioanalytes such as cholesterol,^63^ bilirubin,^64^ and glucose,^65^ among others. These studies show the possibility of using PPy in the facile and low-cost fabrication of point-of-care (POC) and/or wearable devices for various diseases without compromising detection capabilities, as exhibited by the sensor performances compared to standard ELISA techniques.

### 2.3 Poly(3,4-ethylene dioxythiophene)

PEDOT is one of the most widely studied conducting polymers due to its stability and high conductivity, whose highest values can reach up to 6259 S cm^–1^ for thin films^66^ and 8797 S cm^–1^ for a single-crystal nanowire.^67^ PEDOT can be synthesized through three main polymerization routes: oxidative chemical polymerization from EDOT-based monomers using various oxidants, electrochemical polymerization in a three-electrode setup in the presence of an EDOT-based monomer, and transition-metal-mediated coupling.^67−70^ This Review will not go through these in detail, as they have been previously reviewed extensively. PEDOT is an insoluble polymer; hence, it is often mixed with other polymers to enhance its solubility, thereby improving its processability. The most commonly used material for this is poly(4-styrenesulfonate) (PSS), which makes PEDOT more stable in aqueous environments. As a highly biocompatible polymer, PEDOT and its variations such as PEDOT:PSS have been applied to various biomedical fields such as bone, cardiac, and neural tissue engineering^71,72^ and drug delivery.^73^

Because of its combination of excellent biocompatibility and electrical properties, there has been an increase in the use of PEDOT in biomedical sensors and bioelectronics. In biosensors, PEDOT:PSS, combined with graphene oxide (GO), was used as a conductive matrix for the immobilization of glucose oxidase (GOx) for an enzyme-based glucose sensor.^74^ More recently, PEDOT:PSS and GO were also deposited on gold microelectrodes, resulting in the enhancement of their electrochemical, biochemical, and mechanical properties for neural implant applications.^75^ PEDOT was also electrochemically deposited on Mg microwires as biodegradable microelectrodes for neural recordings.^76^ The PEDOT-coated Mg microwires were spray coated with poly(glycerol sebacate) (PGS) as an insulating layer. The performance of the resulting microelectrode was that of comparable platinum (Pt) microelectrodes, which are widely used in the clinical setting. The resulting electrode had improved electrical properties compared to the Pt electrode due to the presence of the PEDOT coating. Specifically, its charge storage capacity was five times that of the Pt electrode and a lower impedance between 1 MHz and 0.1 Hz. Moreover, the Mg-based electrode showed comparable neural recordings in vivo and remained robust after the recordings. Fully organic implantable devices have also been developed based on PEDOT. For example, the group of Ferlauto fabricated a fully organic transient neural probe based on polycaprolactone (PCL) as the substrate and encapsulation material and PEDOT:PSS-ethylene glycol (EG) as the functional electrode material.^21^ The electrodes were implanted in the visual cortex of mice and were used to measure neural activities at rest and upon induction of epileptic seizures and visual stimuli. The authors showed that the electrodes produced good readings months after implantation, although only a few electrodes remained intact three months postimplantation. PEDOT:PSS is not known to be bioresorbable, but the authors hypothesized that the electrode deterioration was due to hydrolysis in relation to its weak adhesion to PCL and to the presence of hydrogen peroxide in the environment. The authors also hypothesized that, compared to a nontransient polyimide probe, the transient probe resulted in a less tight glial scar, which gave the microglia access to the probe to phagocytose the delaminated PEDOT:PSS. Finally, the results showed a complete degradation of the electrode after 1 year at 37 °C at pH 12, with an acceleration factor of about 2.5 compared to pH 7.4. This slow degradation was also demonstrated upon implantation of the electrode with only a minor glial scar after nine months. In another study, Pradhan et al. developed a fully organic, biocompatible, and bioresorbable temperature sensor made of silk and poly(3,4-ethylenedioxythiophene): polystyrenesulfonate (PEDOT:PSS).^25^ Silk is a naturally occurring protein that is mainly obtained from silkworms. It is mainly composed of silk sericin (SS) and SF, which comprises about 75% of silk.^77^ SF has been shown to be biocompatible and biodegradable and has been approved by the US Food and Drug Administration for use in certain medical devices.^78^ On the other hand, SS is the unutilized byproduct of the silk industry, as it is removed from SF to improve aesthetic properties.^79^ However, like SF, SS has been shown to be bioinert and biodegradable and has been used as substrate material in tissue engineering, drug delivery systems, pharmaceuticals, and cosmetics. In the work by Pradhan et al., the substrate and sheathing material of the sensor were made of photoreactive silk fibroin (photofibroin), while the conducting layer was made of PEDOT:PSS dispersed within photoreactive SS (photosericin).^25^ Through the dispersion of the PEDOT:PSS within the biodegradable sericin, its degradation could be controlled. The full sensor could be fully degraded after 10 days in a 3.5 U mL^–1^ protease solution in PBS. The same group also employed the same technique for the fabrication of a silk and PEDOT:PSS-based sensor for glucose, DA, and ascorbic acid.^24^

### 2.4 Natural Semiconductor Analogues

Melanin is a group of phenolic polymers that are naturally found in organisms and has been found to have semiconducting properties.^80^ A study by Mostert et al. has shown that the electroconductivity of melanin stems from its hybrid behavior as an electronic–ionic conductor and not due to its amorphous organic semiconductor nature, as previously thought.^81^ Natural melanins are difficult to extract and often undergo alterations from processing; hence, synthetic analogs are the better options for further investigation. One of the most frequently used precursors for synthetic melanin is DA. DA is used as the precursor for the melanin-type polymer PDA. Aside from its semiconductive property, PDA has a similar structure to the essential component of the adhesive in mussels, resulting in PDA having excellent adhesive properties. Several studies have also shown the biocompatibility of PDA^82^ and the biodegradability of melanin-based films^83^ and hence the possibility to use melanin in bioresorbable and implantable sensors.

Youn et al. used eumelanin, a naturally occurring form of melanin, to impart conductivity to a silk-based film.^84^ The resulting film had a tensile strength of up to 28.4 MPa and could reach a charge storage capacity of 0.3 mC cm^–2^. In addition, the film retained 90% of its electroactivity after 7 days in PBS and 83% of its activity after 100 redox cycles and exhibited no cytotoxicity after 72 h of incubation with L929 murine fibroblasts.

DA and PDA have also been used as dopants to other conducting polymers. For instance, Chalmers et al. used PDA as a dopant to increase the conductivity and the adhesion of PPy hydrogels.^85^ The PDA-doping of the PPy hydrogel resulted in a 2720% increase in conductivity (up to 0.005 S cm^–1^ in the presence of tris buffer) and a 2140% increase in adhesion compared to the undoped PPy hydrogel. The presence of the tris buffer helped with the even distribution of the stiff PDA within the PPy hydrogel, leading to a higher conductivity and softer hydrogel. This was exhibited by a decrease in the Young’s modulus (from 2.1 to 1.1 Pa) of the PDA-doped PPy hydrogel in the presence of tris compared to the increase in the Young’s modulus (from 2.2 to 5.1 Pa) of the PDA-doped PPy in the absence of tris. Zeng et al. also used DA to dope PEDOT:PSS films.^86^ In this work, the PEDOT:PSS film self-assembled upon the mixing of PEDOT:PSS and a DA–hydrochloride solution. The doping resulted in a 10-fold increase in the conductivity of the bare PEDOT:PSS from 9.04 × 10^–4^ to 3.78 × 10^–3^ S cm^–1^ for the doped PEDOT:PSS. In another study, DA was added to PANI, resulting in an increase in the adhesion of the DA-PANI film to a gold substrate.^82^ The results showed a decrease in delamination between the film and the substrate with the increasing DA concentration. However, contrary to the results of the previous two studies, the addition of DA to PANI resulted in a decrease in the conductivity of the PANI film. The authors hypothesized that this could have been due to a disruption of the PANI chains by DA.

## 3 Hydrogels

The first time that the word “hydrogel” appeared in a publication/paper, was in a 1984 publication by Lee et al.^87^ However, the description of that hydrogel differs from the actual definition, as it was a colloidal gel made with inorganic salts.^88^ In 1960, a poly(hydroxyethyl methacrylate) (pHEMA) hydrogel was developed by Wichterle and Lim for use in permanent contact applications in human tissues.^89,90^ Since that moment, the number of papers and references has increased exponentially (Figure 1).

Hydrogels are highly hydrophilic three-dimensional polymeric materials capable of holding large amounts of water (>90%).^91,92^ Among them, those exhibiting biocompatibility and biodegradability have attracted attention in the last decades due to their excellent intrinsic properties, including sensitivity to external stimuli and tunable mechanical properties. Because of these unique properties, they have been used in many applications such as controlled drug delivery systems,^91^ biosensors,^93^ or tissue engineering scaffolds.^94,95^

There are many procedures to synthesize hydrogels depending on their final desired properties. In general, these procedures can be divided into two main groups: physical and chemical cross-linking. The first one includes interactions between polyelectrolytes and polyvalent surfactants/ions. On the other hand, chemical interactions depend on the covalent cross-linking of their polymer structures, which include radical polymerization, grafting, thermogelation, enzymatic reactions, and radiation cross-linking. In addition, as hydrogels contain many functional groups (hydroxyl, carboxyl, amine, and amide), their mechanical and physicochemical properties can be easily tuned. These properties can include swelling ratio, degradation rate, elasticity, viscosity, and stiffness.^96^

### 3.1 Natural Hydrogels

Natural hydrogels are biocompatible, biodegradable, and bioactive materials suitable for biomedical applications. This kind of material presents some advantages compared with synthetic hydrogels, such as higher biocompatibility, better cell adhesion, etc.^97^ However, natural hydrogels present some weaknesses, such as the limited possibilities for their mechanical properties to be tuned and modulated, which means that their potential use is reduced to a few biomedical applications.^98^ Depending on the origin of the polymer, natural hydrogels can be divided into protein-based, polysaccharide-based, and nucleic acid-based materials.^99^

#### 3.1.1 Protein-Based Materials

Protein-based materials are polypeptide units covalently bonded by amide groups. The conformation depends on protein-level structures: primary, secondary, tertiary, and quaternary. The primary level structure is the sequence of amino acids, while the secondary level introduces the orientation of the final structure such as helices, loops, and turns. The tertiary conformation introduces some domains to describe remarkable regions of the AAs, which follow a similar trend. Finally, the quaternary conformation is the description of functional protein formed via multiple polypeptide chains. Naturally occurring protein hydrogels commonly used are collagen, gelatin, and elastin.

**Figure 3:**
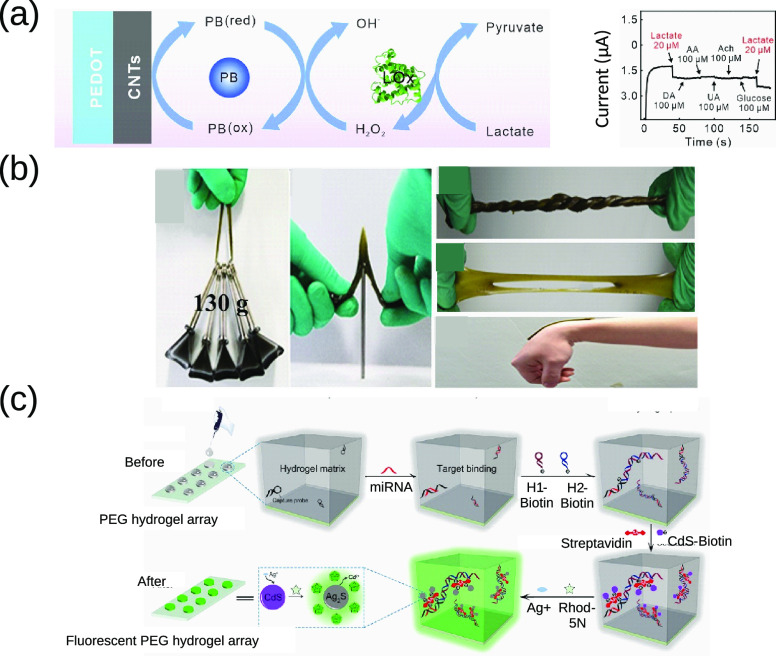
Examples of smart hydrogels adapted from various sources. (a) CPH-based cell culture matrix with lactate sensing capabilities. The images demonstrate the schematic of the lactate detection and the amperometric response of the system with 20 μM lactate and 100 μM interfering agents. Reproduced from ref (100). Copyright 2022 Elsevier. (b) Self-healing and adhesive hydrogel-based strain sensor. The images depict the excellent mechanical properties of the hydrogel, which is shown to be capable of bearing heavy weights and enduring different mechanical stresses. Reproduced from ref (101). Copyright 2020 Elsevier. (c) A highly sensitive microRNA sensor based on a fluorescent hydrogel. The image depicts the mechanism of microRNA detection via interfacial cation exchange fluorescence amplification. Reproduced from ref (102). Copyright 2019 Elsevier.

Collagen is the most abundant extracellular matrix (ECM) protein whose function is to give mechanical support to plastic deformation. The collagen structure consists of a triple helix that gives tensile strength. An excess of cross-linking of the collagen structure gives stiffness to the material. In 2018, Ravichandran et al. published a proof-of-concept biosensor for in vitro glucose detection and monitoring made of a collagen-based electroconductive hydrogel sensor. This soft wearable electrode can be adapted to any tissue and provides a signal response to redox species via enzymatic catalysis.^103^ In another study, Yao et al.^100^ fabricated a 3D enzymatic electrochemical sensor to detect H_2_O_2_ as subproduct from the oxidation of lactate (Figure 3a). This sensor was doped with electrodeposited Prussian blue NPs and carbon nanotubes adsorbed onto the electrode. Lactate oxidase enzyme was then deposited on to the surface. The final electrode was integrated into collagen hydrogel with C6 glioma cells to detect lactate.

Gelatin is derived from denaturing the triple helix of collagen or a mixtures of proteins acquired by the acid or alkaline (pH 3–11) hydrolysis of collagen. The mechanical property of gelatin-based hydrogels can be tuned by adding cross-linking agents, UV radiation, or thermal treatment.^104^ Gelatin is a water-soluble hydrogel, whose most important properties include its emulsifying and foaming capacity, jellification capacity, and use as a thickening material. On the other hand, elastin is a well-known natural hydrogel whose highly cross-linked nature provides robust elastic fibers to tissues and organs. Elastin is an insoluble, chemically inert polymer that can be synthesized using tropoelastin as the basic building block. Elastin hydrogels present high porosity and cross-linking.^105,106^

Both these protein-based hydrogels have huge potential to be used in tissue regenerative technology, drug delivery, and implants, since they both present common properties such as low cost, high availability, biocompatibility, and biodegradability.

#### 3.1.2 Polysaccharide-Based Materials

Polysaccharide structures are based on carbohydrate units covalently bonded via glycosidic groups. Polysaccharide-based hydrogels present good flexibility, low toxicity, high stability, and biodegradability. However, polysaccharides present a lower biocompatibility compared with protein-based hydrogels. The most remarkable polysaccharide polymers used are alginate, cellulose, and chitosan.^107^

Alginate is a polysaccharide composed of two monomers, α-l-guluronate and β-d-mannuronate, organized in blocks and extracted from certain bacteria and brown algae. Alginate presents non-biodegradable and low toxicity properties. Indeed, its hydrogels present better stability and compatibility for tissues and biological fluids compared with other natural-based hydrogels such as chitosan or hyaluronic acid. For example, Wang et al.^108^ developed a sodium alginate/PAM composite hydrogel with glucose oxidase (GOx) to detect glucose in honey and a mineralizer via electrochemical impedance spectroscopy (EIS) measurements at a wide range of temperatures. The purpose of the 3D composite hydrogel was to introduce chemical stability to the sensor.

Chitosan is a linear polysaccharide based on d-glucosamine and *N*-acetyl-d-glucosamine randomly dispersed throughout the network. Mechanical properties of these hydrogels are directly related to their molecular weight and the ratio of deacetylated chitin. Chitosan hydrogels have been widely used in various biomedical fields such as tissue engineering, drug release, and enzyme immobilization and sensing thanks to their shape-memory, self-healing, and self-assembly capabilities.^109^ Chitosan presents antibacterial activity, biocompatibility, and biodegradability but poor mechanical properties. Recently, in 2023, Yin et al.^110^ fabricated an amperometric sensor made of a chitosan hydrogel modified with carbon fiber microelectrodes to detect and monitor glucose, DA, and ascorbic acid. The chitosan hydrogel was used was to avoid nonspecific biomolecule adsorption. The authors observed the preservation of the sensitivity of up to 90% after the passivation of the microelectrodes with the chitosan hydrogel.

Cellulose is a glucose-derived natural polymer whose structure makes it an excellent material for use in sensor applications. Baretta and co-workers published flexible screen-printed electrodes based on Prussian blue NPs with enzymes adsorbed onto the surface.^111^ They fabricated a composite based on a functionalized carboxymethyl cellulose porous hydrogel with the Prussian blue NPs. The hydrogel provided mechanical stability to the electrode and preserved the electrochemical activity of the enzymes attached onto the electrode. This sensor presented a higher sensitivity toward NADH, glucose, and ethanol in serum.

#### 3.1.3 Nucleic Acids

Nucleic acids, such as DNA and RNA, are one of the most discussed biomolecules found in cells. In addition, they present the possibility of forming hydrogels, whose most relevant properties are porosity, biocompatibility, analytical programmability, and high signal sensitivity. Depending on the analyte signal to be recognized, different specific self-assembled DNA or RNA hydrogels can be easily synthesized through their sequence design. DNA-based hydrogels have great potential to be used in biosensing, as DNA can easily recognize a specific target and its subsequent signal reported.^112^ For example, Zezza and co-workers published a copolymerization between methacrylate and thiol-modified DNA via a thiol–epoxy coupling reaction. Acrydite-modified DNA was then used as a reactant to fabricate the hydrogel network with the acrylamide monomer. DNA immobilization was done by click photochemistry. Various DNA-based hydrogels sensitive to different analytes can be fabricated due to the adaptable nature of DNA and its easy sequencing.^113^

### 3.2 Synthetic Hydrogels

Synthetic polymeric hydrogels are 3D networks of hydrophilic polymers or copolymers that are covalently or ionically cross-linked. Synthetic polymeric hydrogels can be divided into different groups depending on their chemical or physical composition. The mechanical properties of synthetic polymer can be easily tuned by changing the chemical composition during the fabrication process. Compared with natural hydrogels, synthetic hydrogels present some improvements related to their mechanical strength, slower degradation rates, and chemical strength. Synthetic hydrogels can be based on poly(acrylates), poly(acrylamides), poly(ethylene oxide) (PEO), and PVA, among others.^114,115^

Cross-linked networks of PAM and its derivatives present interesting temperature and pH sensitivity. Two of the most well-known thermosensitive PAM hydrogels are poly(*N*-isopropylacrylamide) (PNIPAm) and poly(*N*,*N*-diethylacrylamide) (PDEAAM).^97^ These external stimuli properties will be discussed in subsequent sections (see sections 4.1 and 4.2). In addition, PAM can be mixed with other polymers to create copolymers, thereby modifying their properties. For instance, Gao and co-workers published an adhesive and self-healing copolymer hydrogel based on gelatin enhanced with poly(acrylamide-*co*-dopamine) doped with catechol Fe^3+^ (Figure 3b). Catechol Fe^3+^ was added to the hydrogel as cross-linking points creating a synergistic effect between the hydrogel and catechol–Fe^3+^. This complex composite improved the self-healing efficiency of the hydrogel. This hybrid material presented excellent mechanical properties to be used as wearable strain sensors to monitor human body motions.^101^

Acrylate-based hydrogels are some of the most promising biocompatible hydrogels that are used as platforms in many fields, such as therapeutics, drug delivery, and biosensors. The most commonly used acrylate-based hydrogels are synthetic polymers such as PVA, poly(vinylpyrrolidone) (PVP), and poly(acrylic acid) (PAA). PVA is a hydrophilic synthetic polymer that has been used for a long time for biomedical purposes thanks to its nontoxicity and good mechanical resistance. Its use in different applications, such as cartilage replacements and nerve guides,^91^ means that its biocompatibility and bioresorption have been well established.^116^ In addition, its high flexibility and stretchability makes it a good candidate for biosensors. In 2017, Tayebi groups published a composite based on PVA and Fe_3_O_4_ magnetic NPs to fabricate a sensitive glucose sensor. The composite was used as platform to incorporate a GOx enzyme. The sensor was tested by cyclic voltammetry in which LOD was 8 mM.^117^

PEG is one of the most popular polymers to be used in hydrogels thanks to its properties including biocompatibility, good mechanical strength, and natural degradability. Makhsin et.al.^118^ fabricated two different copolymers, PEGDA and poly(ethylene glycol) methyl ether acrylate (PEGMEA), that were functionalized with NH_2_ to improve their sensitivity, since biomolecules can be covalently bonded to amine groups. These were then used to fabricate an optical waveguide sensor. The detection of different biomolecules can be monitored through the refractive index of the hydrogel. In 2022, Park et al.^119^ published microfluidic devices made of a PEG hydrogel composite with platinum nanozyme to detect glucose, which was then inserted into microfluidic channels. The final biosensor was tested using serum, urine, and saliva samples and demonstrated a highly sensitive signal toward glucose and long-term reproducibility.

## 4 Smart Hydrogels

**Figure 4:**
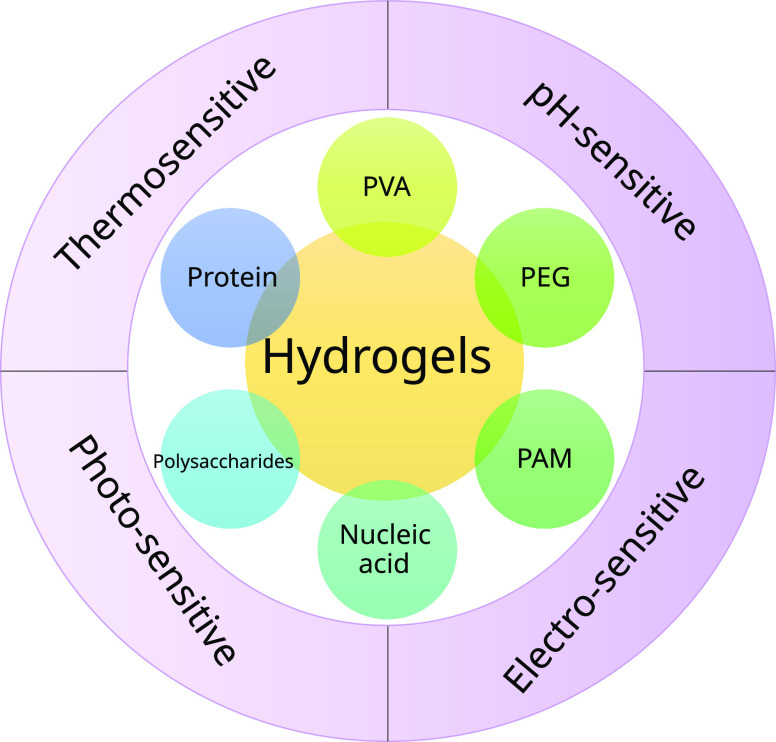
Classifications and types of smart hydrogels.

Hydrogels that exhibit some response to external stimuli are an important source of applications due to their “smart” behavior. Their classification based on their raw materials and response to external stimuli can give a glimpse of their potential applications (Figure 4).

### 4.1 Thermosensitive Hydrogels

Thermosensitivity is the ability of a hydrogel to swell or shrink when temperature is applied, which means that the swelling behavior depends on the environment temperature.^120^ Thermosensitive hydrogels can be divided into three groups: negative temperature, positive temperature, and thermoreversible. Negative-temperature hydrogels exhibit a lower critical solution temperature (LCST), resulting in the hydrogel shrinkage with an increase in temperature. On the other hand, positive-temperature hydrogels have an upper critical solution temperature (UCST) such that these hydrogels solubilize at higher temperatures. Among them, poly(*N*-isopropylacrylamide) (PNIPAm), an intelligent thermosensitive hydrogel, has become essential due to the development of multiple applications in diverse fields such as biomedicine.^121^ For instance, Ross et al. published a chitosan hydrogel cross-linked with PNIPAm to be use for ketotifen fumarate eyedrops in the treatment of allergic conjunctivitis.^122^ Another study recently published by Lanzalaco and co-workers presented a poly(*N*-isopropylacrylamide-*co*-N,*N*′-methylene bis(acrylamide)) hydrogel covalently bonded to a mesh surface. The aim of this study was to functionalize a surgical mesh with a thermosensitive hydrogel to repair hernia defects.^123^ They demonstrated the first 3D movement of a flat surgical mesh bonded with the thermosensitive hydrogel that has the potential to repair hernia defects under human temperature and humidity conditions. Similarly, it was shown that the thermosensitivity of a DNA-anchored PNIPAm hydrogel can act as a good transporter of substances through the thermostimulated “inhalation–exhalation” cycles of the hydrogel matrix. The flow of water through the macroporous channels of the hydrogel due to thermally activated shrinking/swelling cycles, helped to accelerate the entry of external substances and the expulsion of endogenous substances. These new materials with improved transport properties will help in the development of more effective biosensing systems.^124^

### 4.2 pH-Sensitive Hydrogels

As polymers can contain acidic groups, hydrogels can be sensitive to pH changes due to the changes on the hydrophilic–hydrophobic chains. pH-sensitive polymers are named polyacids or polyanions. An example of a pH-sensitive polymer is poly(methacrylic acid) PMMA. The sizes of these gels respond to environment pH and as well as the salt concentration. An equilibrium model was established by Moore’s group to predict the swelling/deswelling behavior of hydrogels in different pH solutions. This model was used to study how the mechanical properties of a hydrogel can be changed in the presence of different buffer solutions.^125^ An optical pH sensor based on a smart hydrogel material was recently designed for use in microfluidics applications. Specifically, a chitosan-tetraethyl orthosilicate (chitosan-TEOS) interpenetrating network was used as a pH sensing element and integrated into a chip. Changes in pH influenced the degree of swelling of the hydrogel, which was monitored by optical elements without the requirement of dyes or additional fluorescent materials.^126^

In a paper published by Yang et al.,^127^ 2-methylacrylic acid-modified cyclodextrin was copolymerized with 2-methylacrylic acid and *N*,*N*-methylene diacrylamide to fabricate a pH sensitive hydrogel. This 2-methylacrylic acid-modified cyclodextrin-based hydrogel was both temperature- and pH-responsive. The authors took advantage of these properties to use the composite as a platform to release atorvastatin, which could protect the stomach against an acidic environment. In addition, they also observed that the hydrogel exhibited significant performance in drug loading and drug adsorption in the three-dimensional hydrogel scaffold, which means that this scaffold could have a huge potential in oral medicine.

### 4.3 Electrical Signal-Sensitive Hydrogels

Electrosignal hydrogels are smart hydrogels that react against an external electric field. These polymers can release or store charge through redox processes.^128^ For example, Neumann and co-workers published a hydrogel based on methacrylic acid (AA) and methyl methacrylate (MMA). This electrical sensitive hydrogel was used as a stimuli-responsive drug release platform.^129^

Das et al. fabricated a urea biosensor in which aniline was electropolymerized within a hydrogel of polyacrylamide (PAM) and PVA. The resulting hydrogel membrane was used to immobilize urea on the surface, which was detected by electrochemical measurements.^130^ This biosensor presented a good linearity of urea concentration, a low detection limit (60 nM), and a high sensitivity (878 mA). They also tested the sensor in real samples, such as milk, blood, and urine, with good results comparable with those of commercially available sensors.

A very different biosensor was recently proposed by Xiong et al.^131^ based on a monolithically integrated hydrogel ion diode for the electrical quantitative detection of DNA. This was made possible by a heterojunction made of adjacent segments of polycationic and polyanionic hydrogels on a microfluidic chip. This device allowed the evaluation of a PCR assay amplifying a 500 bp DNA fragment of *Escherichia coli*. This kind of novel device would facilitate the creation of miniaturized optics-free platforms to quantify nucleic acid amplification at the point of care.

### 4.4 Photosensitive Hydrogels

Photosensitive hydrogels are polymeric networks within which photoreactive molecules such as chromophores have been incorporated. These kinds of hydrogels can modify their structure and conformation according to light or dark conditions. To synthesize these hydrogels, some photoresponsive functional groups must be added to the polymer chain. This response can be reversible or not depending on the chromophore.^132^ Some groups have been working on these properties to fabricate biosensors. For instance, Wu et al.^102^ presented a fluorescent PEG hydrogel with high sensitivity to microRNA based on cation exchange coupled with DNA hybridization, wherein the microRNA bonded to the carboxylate groups of PEG. Once the DNA strand was attached to the hydrogel, biotinylated CdS quantum dots (QDs) were bound onto DNA strands in a sandwich format (Figure 3c). A mixture of Ag^+^ and rhodamine-5N dye was then introduced into PEG matrix. The idea of the cation exchange between Ag^+^ and rhodhamine-5N was to amplify the fluorescence intensity from the semiconductor, thus increasing the quantum yield of CdS QDs. This fluorescence composite presented a LOD down to 0.835 fM.

Li’s group presented a proof of concept of the fabrication of a cathodic photoelectrochemical enzymatic biosensor based on a PANI hydrogel and donor–acceptor active material PTB7-Th to detect and quantify guanine.^133^ As mentioned in earlier sections, PANI presents excellent electrical properties and good compatibility, making it suitable to be used as a biosensor platform. The purpose of coating PTB7 on to PANI-glassy carbon electrode (GCE) was to improve the sensor’s current response. This photocathodic enzymatic biosensor presented a LOD of 0.02 μM and a wide linear range from 0.1 to 80 μM.

## 5 Conducting Polymer Hydrogels

**Figure 5:**
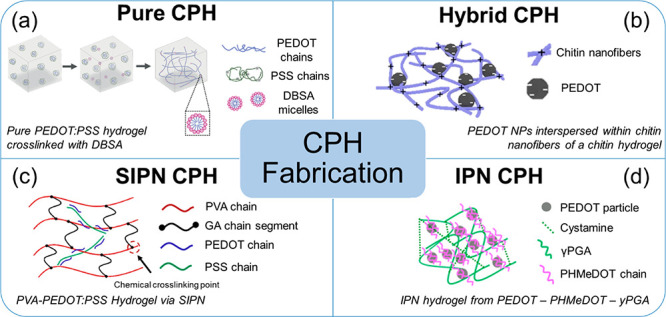
Illustrations of different CPH fabrication types. (a) Pure CPH. Reproduced from ref (138). Copyright 2020 Wiley. (b) Hybrid CPH. Reproduced from ref (139). Copyright 2021 American Chemical Society. (c) Semi-interpenetrating polymer network (SIPN) CPH. Reproduced from ref (140). Copyright 2020 Elsevier. (d) Fully interpenetrating network (IPN) CPH. Reproduced from ref (141). Copyright 2021 Wiley.

CPHs are conducting polymers contained in a supporting polymer hydrogel. CPHs can be either fabricated from pure CPs or from the incorporation of conduction polymers into a supporting hydrogel. However, one of the main advantages of the use of pure CPHs is that the intrinsic electroactivity of the CPs is not decreased by mixing them with nonconductive additives. On the other hand, other important features such as biodegradability and mechanical properties cannot be enhanced without the addition of other polymers. Hence, the CPHs are more commonly found as conducting polymers incorporated in a second polymer. This incorporation can be a simple dispersion (hybrid CPH), a semi-interpenetrating network (SIPN), or a fully interpenetrating network (IPN) (Figure 5). In addition, smart hydrogels can be processed in different geometries and modalities to meet the complicated situations in biological media, namely, injectable hydrogels (following the sol–gel transition),^134^ colloidal nano-^135^ and microgels,^136^ and three-dimensional (3D) systems.^137^

### 5.1 Pure CPH

Pure CPH, or CPH without supporting polymers, is commonly prepared through the use of cross-linkers (Figure 5a). For instance, Zhang et al. fabricated PEDOT:PSS hydrogels at room temperature using 4-dodecylbenzenesulfonic acid (DBSA) as the cross-linking agent, resulting in a self-healing hydrogel with a conductivity of 0.1 S cm^–1^ and a Young’s modulus of 1 kPa.^138^ One of the very first pure conducting polymer hydrogels to be fabricated is based on PANI with the use of phytic acid (PA) as both the gelator and dopant.^142^ In this work, Pan et al. illustrated that PA protonates the nitrogen groups of the PANI and can interact with multiple PANI chains, thereby resulting in a mesh-like structure. The resulting hydrogel could fully cross-link in 3 min and could be purified through several washings. Moreover, this PA-based gelation could also be applied in the fabrication of pure PPy hydrogels using a liquid-phase interfacial reaction^137^ as well as copper phthalocyanine-3,4′,4″,4‴-tetrasulfonic acid tetrasodium salt (CuPcTs).^143^ On the other hand, Lu et al. reported a simple and inexpensive synthesis of pure PPy hydrogels.^144^ This multistep approach begins with dissolving PPy in a mixture of water and ethanol, followed by oxidation in ferric nitrate as the limiting reagent. The resulting gel was then “aged” for about 30 days to facilitate secondary polymerization to further strengthen the hydrogel.

Despite their ease of use, these cross-linking agents are often nonconductive and thereby reduce the conductivity of the CPH. To address this, other methods have also been developed. For instance, a PEDOT:PSS hydrogel was fabricated through a dry annealing and swelling process with dimethyl sulfoxide (DMSO) as an additive.^145^ The DMSO enhanced not only the conductivity of the hydrogel but also its mechanical properties. The resulting hydrogel had a stretchability of about 35% strain, a Young’s modulus of 2 MPa, and a conductivity of about 20 S cm^–1^ in PBS and 40 S cm^–1^ in deionized water. On the other hand, Feig et al. also proposed an electrogelation method for the fabrication of complex PEDOT:PSS hydrogel structures. In this method, a copper structure was deposited on the working electrode via shadow masking.^146^ The working electrode was then placed in a PEDOT:PSS solution and placed under constant current followed by constant potential, leading to the oxidation of copper into Cu^2+^. Upon the oxidation of copper, the initial repulsion between the colloidal PEDOT:PSS microgel was screened, resulting in gelation. Highly complex structures of PEDOT:PSS hydrogels can be fabricated using this method because the patterning is dependent on copper-patterning, which has been well established. In another study, Guo et al. fabricated a self-cross-linked PANI hydrogel using aniline hydrochloric salt as the precursor and APS as the oxidizing agent via a sol–gel method.^147^ The resulting hydrogel exhibited a specific capacitance of 588 F g^–1^ at the completely doped state and that of 418 F g^–1^ at the fully dedoped state.

### 5.2 Hybrid CPH

Another strategy in the fabrication of CPH is the dispersion of conducting particles within a polymer hydrogel matrix (Figure 5b). Typically, these conducting particles are added in the pregelated mixture and get dispersed within the hydrogel matrix upon gelation. Huang et al. incorporated PEDOT NPs, which were synthesized via chemical oxidation polymerization, into a chitin hydrogel.^139^ The resulting hydrogel was further decorated with the tetrapeptide Cys–Arg–Gly–Asp (CRGD), which facilitated cell adhesion and proliferation. Upon implantation in rats, the hybrid hydrogel was shown to improve sciatic nerve regeneration due to its conductivity and improved Schwan cell adhesion. In another study, PPy nanotubes (PPyNT) were synthesized through the oxidation of pyrrole monomers and dispersed within a PNIPAm precursor.^148^ After the gelation of PNIPAm, the distribution of PPyNT within the hydrogel could be modified at wil, in a matter of seconds, through thermal or illumination changes. Upon illumination or an increase in temperature, a conductive pathway from the nanotubes was formed, resulting in an improvement in charge storage and transport, while the reverse took place when the stimulus was removed. This interesting phenomenon could be of great interest for building smart supercapacitors.

Aside from conductivity, these CP NPs can also be modified to impart other desired properties into the hydrogel. For instance, Gan et al. took inspiration from mussels to fabricate conductive nanofillers, which endowed a polyacrylamide (PAM) hydrogel with both conductivity and adhesiveness.^149^ In this work, the NPs were composed of a specific conducting polymer (PEDOT, PPy, or PANI) and sulfonated lignin, which acted as a CP dopant and a provided a catechol group to improve the hydrophilicity and redox reactions of the CP. The resulting hybrid hydrogel showed improvements in conductivity and adhesiveness compared to bare PAM hydrogels and PAM hydrogels filled with bare CP NPs.

### 5.3 Semi-Interpenetrating CPH

A third method for preparing CPH is through the formation of a semi-interpenetrating polymer network (SIPN). A SIPN is fabricated when linear polymer chains are embedded within a second polymer network (Figure 5c). If these linear chains are further cross-linked, a fully interpenetrating network (IPN) is formed.^150^ For instance, Puiggalí-Jou et al. added a PEDOT:PSS suspension to an alginate mixture, which was then cross-linked with the addition of calcium (Ca^2+^) ions. The resulting hydrogel was electroresponsive and could be used for controlled drug release.^151^ A similar strategy was also employed by adding a PEDOT:PSS suspension in a PVA solution, which was then placed under several freeze–thaw cycles to obtain a physically cross-linked conductive PVA hydrogel.^152^ This resulting hydrogel was then used to fabricate electrodes in a gel-based supercapacitor. A PVA–PEDOT:PSS SIPN hydrogel can also be fabricated using glutaraldehyde (GA) as the chemical cross-linker for PVA.^140^ On the other hand, a zwitterionic SIPN based on PANI and a copolymer of anionic acrylic acid (AA) and cationic methylacryloyl oxygen ethyl trimethylammonium chloride (DMC) was fabricated as a candidate for flexible strain sensors.^153^ In this work, the hydrogel from p(AA-*co*-DMC) was first fabricated, followed by the polymerization of aniline using APS. The resulting hydrogel can reach a tensile strength of 0.173 MPa, a strain of 576%, and a conductivity of up to 0.004 S cm^–1^. It also demonstrated quick and reproducible responses to human motions, such as joint and throat movements. The presence of the interspersed secondary network can improve not just the conductivity of the hydrogel but also the mechanical strength. Such was the case for the SIPN network based on PPy and acrylic acid-modified nanocrystal cellulose (AA-*g*-NNC).^154^ In this work, polymerization of PPy took place within the AA-*g*-NNC hydrogel matrix using ferric trichloride and sodium *p*-toluenesulfonate. The resulting conductive SIPN hydrogel exhibited about 4-fold increase in the swelling ratio, an 18-fold increase in compression modulus, and increase from a break stress of 1.53 MPa at a strain of 50% to a break stress of 13.8 MPa at 80% strain. The SIPN strategy can also be employed between a CP and a supporting hydrogel consisting of a multiple network. This was the case with the study conducted by Azar et al., wherein PEDOT:PSS formed a SIPN with a triple network of agarose, PVA, and PAM.^155^

### 5.4 Interpenetrating Network CPH

Lastly, the formation of IPN between a conducting polymer and a supporting polymer can be used to fabricate CPH (Figure 5d). For instance, an IPN of [PEDOT/γPGA]PHMeDOT was fabricated by Molina et al.^141^ In this work, a hybrid CPH was first fabricated by dispersing PEDOT particles within a poly-γ-glutamic acid (γPGA) hydrogel. The resulting conductive hydrogel was then immersed in a monomer solution of hydroxymethyl(3,4-ethylenedioxythiophene) (HMEDOT) and then placed under anodic polymerization. This resulted in the formation of a poly(hydroxymethyl (3,4-ethylenedioxythiophene)) (PHMEDOT) network through the polymerization of the HMEDOT monomers and interconnection with the PEDOT particles. Similarly, a polyaspartic acid hydrogel was used as a base to obtain a conductive IPN hydrogel by loading PEDOT NPs and the subsequent in situ electropolymerization of PHMEDOT.^156^ In another work, an IPN was fabricated based on PANI and poly(acrylamide-*co*-sodium acrylate) (ASH). In this work, a poly(acrylamide-*co*-sodium acrylate) hydrogel was first fabricated through chemical polymerization.^157^ The ASH hydrogel was then swollen, frozen, and thawed to create pores in which aniline could penetrate. Next, a PANI network was synthesized through the use of PA. The final IPN CPH exhibited a conductivity of up to 0.004 S cm^–1^ and a tensile strength of up to 0.12 MPa. On the other hand, Li et al. utilized the IPN strategy to fabricate a “triple network” CPH based on PPy, PVA, and PAA.^158^ In this work, the group first synthesized a PPy nanotube sponge through the chemical polymerization of PPy. The sponge was then placed in a solution containing PAA and PVA and placed under vacuum to allow the infiltration of PAA and PVA. Finally, the PVA was cross-linked through freeze–thaw cycles and PAA using ferric ions. The resulting hydrogel of the PAA, PVA, and PPy nanotube network exhibited a conductivity of up to 0.04 S cm^–1^ and fast responses to compression changes.

## 6 Applications of Conducting Polymer Hydrogels in Biological Sensors

Because of their advantages and versatility, CPHs have been used in various biomedical sensors. In the following sections, recent works on the use of CPHs in diagnostic, wearable, and implantable sensors will be highlighted. These studies include works that use the CPH as part of a sensor device as well as works that exhibit their potential use in a sensor device.

### 6.1 CPH in Biosensors

**Figure 6:**
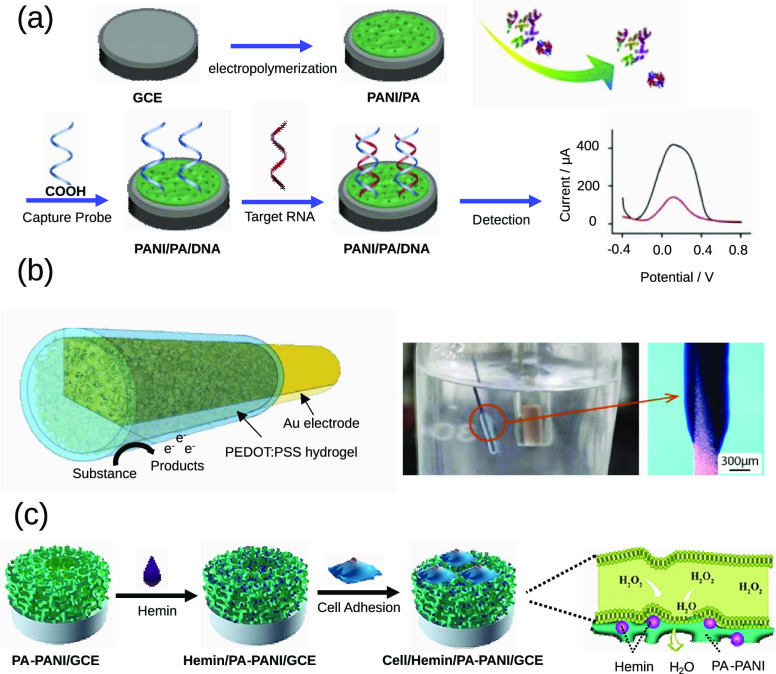
Examples of CPH-based biosensors adapted from various sources. (a) MicroRNA detector based on a PANI/PA hydrogel. Reproduced from ref (14). Copyright 2020 Elsevier. (b) Gold electrodes coated with a PEDOT:PSS hydrogel via electrogelation used as a glucose, hydrogen peroxide, DA, and l-DOPA sensor. Reproduced from ref (13). Copyright 2022 Elsevier. (c) PANI hydrogel doped with PA and hemin as a sensor for hydrogen peroxide from living cells. Reproduced from ref (159). Copyright 2021 Wiley.

Yang et al. developed a PANI hydrogel-based sensor for microRNA.^14^ Specifically, a PANI–PA hydrogel was cross-linked on the surface of a GCE using 3-aminophenylboronic acid and then covered with a DNA probe (Figure 6a). The hydrogel-based platform exhibited excellent selectivity, sensitivity, and reproducibility, as well as a six-day stability of sensing microRNA, as the hydrogel provides a good environment to maintain the bioactivity and high affinity of the DNA probes.

Wang et al. developed a hydrogel-based multisensor for the simultaneous detection of DA, UA, and ascorbic acid.^160^ In this work PPy was combined with an azo dye, tartrazine, that acted both as a cross-linker and dopant for PPy. The final PPy hydrogel was used to coat the surface of a GCE. The simultaneous detection of DA, UA, and ascorbic acid could be achieved because the three biomolecules exhibited oxidation peaks at different potentials with the PPy-hydrogel-GCE device, which could not be differentiated with a bare GCE or a PPy-GCE device. The PPy-hydrogel-GCE device showed reproducible results, low detection limits, stable readings after two weeks of storage, and the ability to detect all three biomolecules in a real sample, such as urine. PPy was also used as the conducting polymer in a hydrogel-based DA sensor by Hwang et al.^161^ In this work, the aptamer-coupled PPy was dispersed within an agarose hydrogel, with the PPy and the aptamer endowing the hydrogel with conductivity and selectivity toward DA, respectively. Agarose can be solidified and liquefied reversibly through temperature changes, thereby allowing the retrieval of the PPy aptamers and resulting in a recyclable sensor.

On the other hand, Li et al. employed electrodeposition to coat gold electrodes with a PEDOT:PSS hydrogel for the electrochemical detection of DA, hydrogen peroxide (H_2_O_2_), glucose, and levodopa (L-Dopa).^13^ The hydrogel electrode was soaked in GOx for the detection of glucose and in colloidal gold for the detection L-Dopa and DA, while platinum NPs were electrodeposited into the hydrogel for the detection of H_2_O_2_ (Figure 6b). Scanning electron microscopy (SEM) images showed the uniform dispersion of these NPs within the hydrogel, and chronoamperometry detection demonstrated good sensitivity, low limits of detection, and reproducible readings by the hydrogel-based electrode.

The studies presented here show interesting applications of CPH-based sensors in aqueous environment of the desired analyte and in some cases with the presence of interferents. Hence, although the results are promising, further studies on the use of these sensors on real human samples could provide more insight in their clinical uses.

Aside from biomolecules, CPH-based electrodes have also been employed for the detection of tumor markers. For example, an anticarcinoembryonic antigen was deposited on the surface of a PPy hydrogel.^15^ In this work, cellulose nanocrystals were used as the PPy cross-linkers, and sodium benzenesulfonate was used as a stabilizer and dopant. The resulting CPH-based hydrogel exhibited a detection range from 1 fg mL^–1^ to 200 ng mL^–1^, a very impressive low LOD of 0.06 fg mL^–1^, and reproducible and highly selective measurements in human serum.

Another field of application for CPH is cell culture and tissue engineering, wherein it can be used as an electroconductive matrix capable of promoting cell proliferation and differentiation in various areas such as cardiac,^162^ osteocyte, cartilage,^163^ and neural^164^ tissue engineering. In addition, CPHs can also be used as sensing hydrogel matrices in cell culture applications. For instance, Jiang et al.^165^ deposited a PANI–PA hydrogel, loaded with *N*-(aminobutyl)-*N*-(ethylisoluminol) (ABEI) functionalized silver (Ag) NPs, on a GCE to fabricate an in situ electrochemiluminescent (ECL) H_2_O_2_ sensor. Hela cells were then seeded on the hydrogel and incubated with phorbol 12-myristate-13-acetate for H_2_O_2_ detection. The Ag NPs in the hydrogel catalyzed the H_2_O_2_ and produced hydroxyl radicals, which in turn facilitated the ECL reaction of ABEI. On the other hand, the PANI–PA hydrogel provided a conductive matrix for fast charge transfer, the immobilization of ABEI-Ag, and a biocompatible substrate for cell adhesion. The ECL sensor exhibited a detection range of 0.01–40 μM with an excellent detection limit of 3.3 nM and also good selectivity in the presence of other interfering molecules. Likewise, a similar hemin/phytic acid-doped PANI hydrogel composite was used to fabricate an electrode for another electrochemical in situ detection of hydrogen peroxide.^159^ In this work, instead of using ABEI-Ag, Shang et al. functionalized the surface of the hydrogel with hemin, a natural metalloporphyrin that has a peroxidase-like ability (Figure 6c). In another similar study, sulfonated multiwall carbon nanotubes (MWCNTs) were used as cross-linkers for PPy hydrogels for the fabrication of an in situ DA sensor.^16^ The hydrogel had gold NPs, as catalysts for DA, dispersed within the matrix, then was placed on a GCE and seeded with three different nerve cells. The resulting hydrogel exhibited a conductivity of up to 0.3 S cm^–1^, a DA detection range of 0.05–1100 μM, and a detection limit of 17 nM. Another interesting finding from the work was the cability of the CPH-based sensor for the real-time, noninvasive, and label-free detection of cellular activity based on impedance measurements.

Because of the ethical and clinical translation issues of animal trials, the field of organ-on-chip systems is growing exponentially. Hence, cell culture platforms with in situ sensing capabilities, such as these CPH-based matrices, would be of great interest. However, these long-term stability and functionality of these platforms must be tested to exhibit their applicability in cell culture studies.

Finally, CPH has also been used to develop sensors for other molecules. For example, Gao et al. fabricated a nitrite sensor based on a pure PANI hydrogel incorporating CNTs.^166^ Nitrite is an important molecule in the food industry. In this study, nitrite could be detected via its catalysis by both PANI and CNTs. This nitrite-sensing CPH could be adsorbed well on the surface of a printed electrode, resulting in a flexible electrochemical sensor with a nitrite sensitivity of 254.54 mA mM^–1^ cm^–2^.

### 6.2 CPH in Wearable Sensors

Wearable sensors allow for the constant monitoring of physiological and disease-related signals, thereby reducing the need for hospital trips and diagnostic costs. Because of this, there has been a steady increase in the development of wearable sensors.^167^ Thanks to their biocompatibility, conductivity, and tunable properties, CPs have gained popularity in this field.

**Figure 7:**
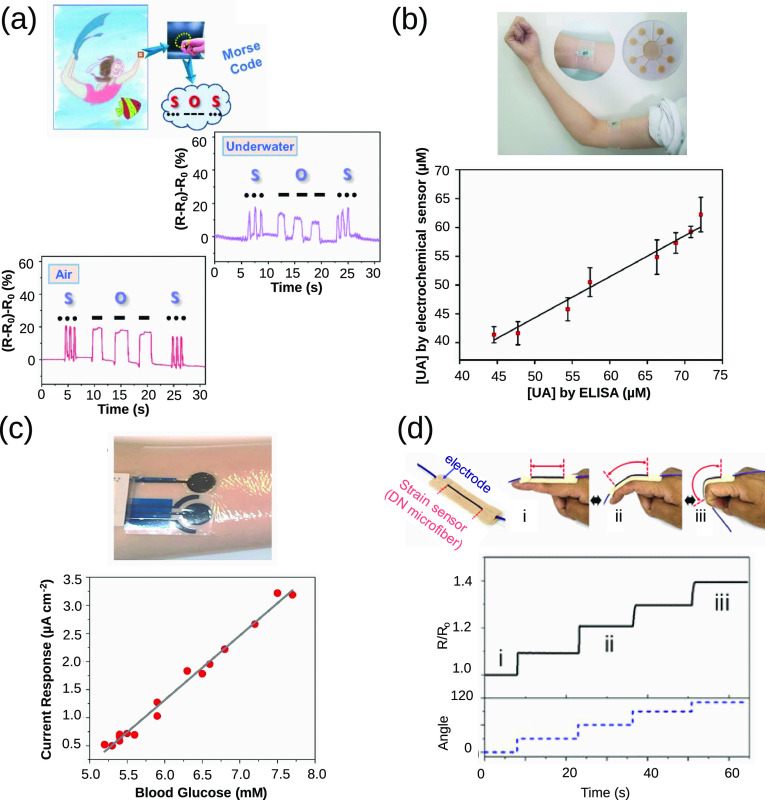
Examples of CPH-based wearable sensors adapted from various sources. (a) Underwater strain sensor. The graphs depict similar resistance changes upon performing the same movements of a finger with the hydrogel attached to it in air and underwater. Reproduced from ref (18). Copyright 2022 Elsevier. (b) Electrochemical UA detector with a microfluidics-based swear collector. The graph depicts a good correlation between the UA concentration measured by the sensor against that measured by ELISA. Reproduced from ref (168). Copyright 2021 Elsevier. (c) Interstitial fluid glucose detector with reverse iontophoresis. The graph depicts a good correlation between the glucose measured on a human subject via the developed sensor and that measured via a traditional glucometer. Reproduced from ref (169). Copyright 2021 Elsevier. (d) Microfluidics-fabricated strain sensor. The graph shows the ability of the sensor to detect various angles via finger movements. Reproduced from ref (170). Copyright 2019 Wiley.

Interruptions in the conductive pathway can cause changes in the resistance of the CPH; hence, several studies have shown the possibility of using CPH in wearable strain and pressure sensors.^171−173^ For instance, Yue et al. developed a hydrogel-based pressure sensor composed of PAA, PEO, and PANI.^17^ The resulting hydrogel exhibited a stretchability of up to 1500% and an elastic modulus retention of 80% after 2000 stretch cycles. Furthermore, the authors also showed the possibility of pressure-based 3D printing the hydrogel without negatively affecting mechanical properties. Finally, a capacitive pressure sensor was constructed from the hydrogel with a sensitivity of 7 kPa^–1^. Similarly, Sun et al. developed a strain sensor based on PAM and a PEDOT:PSS hydrogel incorporated with MAA.^174^ The addition of MAA improved both the adhesion of the hydrogel to various substrates and the conductivity of the resulting hydrogel, as the carboxylic group of the MAA can form hydrogen bonds with PSS. As a proof of concept, the hydrogel was attached to a finger and different English letters were written. Interestingly, different resistance waveforms were obtained for each letter drawn. Similarly, Kim et al.^170^ also fabricated a hydrogel-based strain sensor based on a double network of PAM and calcium alginate microfibers containing PEDOT:PSS (Figure 7d). A unique feature of this CPH strain sensor was that it was fabricated using a microfluidics system in a two-step cross-linking device. In the first step, the pregel mixture was surrounded with calcium chloride solution for the ionic cross-linking of alginate, followed UV irradiation for covalent cross-linking. Zheng et al. also developed a wearable strain sensor with the added application for underwater usage.^18^ The hydrogel-based sensor was composed of silk fibroin (SF), tannic acid (TA), and PPy without the use of any chemical cross-linkers (Figure 7a). The addition of the polyphenol, TA, not only improved the mechanical properties of the hydrogel but also enabled the self-repair and wet adhesion of the hydrogel. The final hydrogel exhibited interesting properties such as self-healing with only a slight loss of mechanical properties and conductivity, adhesivity to various substrates, stretchability up to 500% strain, compliance, conductivity of up to 3.4 × 10^–4^ S cm^–1^, biocompatibility, and antimicrobial properties. As a wearable strain sensor, the hydrogel was attached to different parts of the body and resulted in changes in the relative resistance in accordance with body movements like coughing, frowning, and joint bending. Furthermore, the underwater applicability was also tested by attaching the hydrogel to a finger joint, which was then placed underwater. The change in resistance was only slightly lower when the movements were performed underwater compared to that when movements were performed in air, thus demonstrating the applicability of the hydrogel in underwater wearable sensors. Zhang et al., on the other hand, developed a photocurable hydrogel-based strain sensor composed of a SIPN of PEDOT:PSS and a copolymer of *N*-hydroxyethyl acrylamide (HEAA) and *N*-(3-sulfopropyl)-*N*-methacryloylamidopropyl-*N*,*N*-dimethylammonium betaine (SBAA).^172^ In this work, the hydrogel was a fabricated through a one-pot strategy, wherein PEDOT:PSS in an aqueous solution was mixed with HEAA and SBAA monomers and a photoinitiator. The mixture was then exposed to UV light, causing the photopolymerization of poly(HEAA-*co*-SBAA) and eventual hydrogel formation. The resulting hydrogel exhibited ultrahigh stretchability of up to 5000%, an elastic modulus of up to 0.18 MPa, biocompatibility, and self-healing and antifouling capabilities. Furthermore, a linear response in relative resistance was also obtained with a response time of less than 0.025 s. Zhang et al. developed a strain-sensitive hydrogel that is highly resistant to corrosive solutions.^175^ In this work, the addition of *N*,*N*′-methylenebis(acrylamide) and GO nanosheets into the SIPN of PEDOT:PSS and PNIPAm enhanced the chemical and physical cross-linking of the hydrogel. This in turn rendered the hydrogel more flexible and resistant to various solvents, including toluene, ethyl acetate, and cyclohexane, even at extreme pH levels of 1 and 14, all while keeping its strain-sensing capabilities. Finally, Sun et al. developed a CPH-based wearable sensor, which also exhibited triboelectric properties.^176^ The triboelectric effect happens when opposite charges form on the surfaces of two different materials after coming into contact with one another.^177^ In this work, the group fabricated a triboelectric nanogenerator (TENG) by sandwiching the CPH between silicone rubber and polyurethane (PU), wherein the CPH was based on PEDOT:PSS, gelatin, and PAM. The resulting TENG device with a surface area of 60 × 60 mm^2^ exhibited a current of 26.9 μA, charge of 92 nC, and open circuit voltage of 383.8 V and could power up to 1081 LED arrays in series and an electric watch when combined with a capacitor. As a strain sensor, the device could detect a variety of human motions when attached to the fingers, face, and knee joint and exhibited a gauge factor of up to 1.58 at up to 2850% strain. Finally, Yang et al. developed a skin-compliant hydrogel based on gelatin incorporated with sandwich sheets of PPy and reduced graphene oxide (rGO) sensitive to multiple factors.^178^ The sandwich layer, which was composed of three layers (PPy-rGO-PPy), was fabricated via solvothermal synthesis and was incorporated into physically and chemically cross-linked gelatin hydrogels. This incorporation not only endowed the hydrogel with conductive paths but also improved its mechanical properties through the physical entanglement of the sheets within the matrix. The resulting hybrid hydrogel exhibited a tensile strength of up to 110 kPa and up to 400% strain at the highest PPy-rGO-PPy content, as well as only a slight decline in tensile strength until 200% strain after 20 cycles. As a sensor, the hydrogel exhibited a conductivity of up to 0.8 S cm^–1^, a strain gauge factor of 1.98 within 0–200% strain, a pressure sensitivity of −3.57% kPa^–1^ at pressures below 10 kPa, and a temperature coefficient of resistance (TCR) of 1.29% °C^–1^ under the normal body temperature range of 35–40 °C.

Other previous works on wearable CPH-based pressure and strain sensors are summarized in Table 1. Based on the number of studies, it is evident that there is great applicability for the use of CPH in wearable strain and pressure sensors. These studies differ in the materials used and fabrication process, providing the sensors with different properties. Hence, these sensors can be used in a wide range of applications such as underwater, in the presence of corrosive chemicals, or even at extreme temperatures. Aside from applications, these sensors also differ in their sensitivities and strain and pressure ranges. For these reasons, the choice of CPH-based wearable strain and pressure sensor would largely depend on the final application.

**Table 1:** CPHs in Wearable Pressure and Strain Sensors

conducting polymer	hydrogel	other material	sensing features	ref
PEDOT:PSS	PHEA^a^	laponite	detection of human movements: fingers, wrist, and abdomen	(179)
PEDOT:PSS	PAM–PVA	CNT	detection of finger movements at different angles	(180)
			detection of pulse signals from human radial and carotid arteries	
			respiration monitoring from mouse chest	
PPy	PAA	TOCNFs^b^	gauge factor (measure of sensitivity of strain sensor): 7.3 with 2 linear regions (0–200%, 300–800% strain)	(181)
			bdetection of cyclic finger movements at different angles	
PEDOT:PSS	PAM-Alginate	NA	detection of finger movements, knee joint movements during walking and running at different speeds	(170)
			monitoring of plant growth	
			linear resistance between 0–300% strain with resolution of 0.1%	
PPy	PAA	TB^c^	detection of finger movements at different angles	(182)
			linear response between 0.06 and 0.14 MPa	
PEDOT:PSS	Alginate	NA	low pressure detection: up to 6% 10^–5^ Pa	(183)
PANI	P(AA-co-DMC)	NA	detection of human movements: fingers and swallowing	(153)
			linear and repeatable resistance change up to 100% strain	
PANI	PU-DA^d^	PEG	detection of finger and wrist movements at different angles and throat motion with different phrases	(184)
			recyclability	
PPy	PAM	NA	gauge factor: up to 2.9 with linear region of 0–500% strain	(185)
			freezing tolerance (up to –20 °C)	
			flame resistance (up to 600 °C)	
PPy	gelatin	PVDF^e^	photodetector: up to 350 Hz light switching frequency, detection of various photointensities	(19)
			pressure sensor: range of 0.1–55 kPa, sensitivity of 32.39 kPa	

a Poly(*N*-hydroxyethyl acrylamide).

b TEMPO-oxidizedcellulose nanofibers (TOCNFs).

c Trypan blue.

d Polyurethane–Diels–Alder bonds.

e Polyvinylidene fluoride

CPH can also be used in wearable biological sensors. For example, Xu et al. developed a wearable hydrogel-based glucose sensor supported on screen-printed carbon electrodes.^169^ This hydrogel sensor was composed of PEDOT:PSS with embedded GOx and Prussian blue NPs and mixed with DMSO and Zonyl FS-300 (ZFS) (Figure 7c). The Prussian blue NPs acted as an artificial peroxidase, while DMSO and ZFS helped produce a homogeneous hydrogel with a higher electrical response. The resulting wearable sensor exhibited two sensitivities of 340.1 μA mM^–1^ cm^–2^ at a lower glucose concentration range and 184.3 μA mM^–1^ cm^–2^ at a higher range due to a change in the enzymatic reaction kinetics at low and high glucose concentrations. The addition of different interferents such as sodium chloride and ascorbic acid resulted in minimal current variation. The device was tested in the presence of rabbit serum and resulted in a relative standard deviation of less than 6%, which is lower than the FDA-approved 20% deviation for clinical blood glucose monitoring systems. In addition, the device was also tested on two human volunteers. In this test, the device was placed on the skin and glucose was extracted via reverse iontophoresis from the interstitial fluid (ISF). The resulting glucose measurements were comparable to those obtained using a finger-stick glucometer.

Another research group also developed a wearable PEDOT:PSS hydrogel-based microfluidic sensor for the detection of UA in sweat.^168^ In this work, PEDOT:PSS was fabricated on screen-printed carbon electrodes using electropolymerization with copper as a sacrificial anode, and the microfluidic device was composed of polydimethylsiloxane (PDMS) to pool sweat (Figure 7b). The amperometric response of the device to UA resulted in a linear response with a sensitivity of 0.875 μA μM^–1^ cm^–2^ within the range of 2–250 μM, which falls well within the range of UA concentrations in human sweat. The microfluidic device was also tested with human sweat, and the UA readings obtained from it had a good Pearson correlation with the UA measurements obtained via ELISA. Moreover, the device retained 95% of its original response after 25 days and showed no significant effect on its electrochemical response under deformation, making it a promising wearable UA sensor.

Existing studies show promising results from the use of CPH-based wearable sensors, as they show the use of the materials not only to detect biomolecules in real human samples but also as part of a functional wearable sensor. However, the consolidation of this type of device should lead to more in-depth studies in the near future that involve more in vivo tests and study of the long-term stability and the effects of the sensor on the skin.

Aside from wearable mechanical and molecular sensing, CPH-based materials can also be used in thermosensitive wearable sensors. For instance, Lo et al. developed a self-sensing photothermally responsive hydrogel based on an IPN between PNIPAm and PPy.^186^ In this work, PPy converts optical energy into heat, which then causes the shrinkage of the thermosensitive PNIPAm and in turn decreases the resistivity of the system due to the compaction of the PPy. Moreover, the hydrogel also exhibited light sensitivity, where it was observed to be directed toward NIR light, and a piezoresistivity up to 200% strain. PNIPAm was also the basis of the thermosensitivity of a CPH developed by Zhan et al. that also exhibited pressure and pH sensitivity.^20^ Specifically, PANI was used as the CP- and pH-sensitive component, while MWCNTs were added to improve the conductivity and stability of the hydrogel. Moreover, the addition of carboxymethylchitosan further contributed to the hydrogel’s mechanical attributes. These studies demonstrate the possibility of a next generation of hydrogel-based materials that could also be applied to soft robotics and skin-like bioelectronics.

Traditional ECG and EMG electrodes are made of Ag|AgCl gel. However, these materials could cause skin problems such as dermatitis^187^ and discomfort due to their bulkiness.^188^ For these reasons, alternative electrode materials are being explored. For instance, Zhou et al. fabricated epidermal patch electrodes based on a PVA–PEDOT:PSS hydrogel.^189^ The resulting hydrogel electrode exhibited self-healing properties, good skin adhesion, and high-quality ECG and EMG readings compared to traditional Ag|AgCl gel electrodes. Moreover, the hydrogel-based electrodes could be safely peeled off from the skin, without causing any irritation after an hour of use. Similarly, Wang et al. also employed a PVA–PEDOT:PSS based hydrogel as an electrode for ECG measurements with the addition of carboxymethyl cellulose into the hydrogel mix.^190^ Similar to the results of Zhou et al.,^189^ the hydrogel-based electrodes also exhibited comparable ECG readings to those of commercial Ag|AgCl electrodes at rest and during exercise and even after 35 days. These works are proof of concept to the use of CPH as alternative materials to traditional ECG and EMG electrodes, though further long-term performance of these electrodes must be investigated.

Finally, another application of CPH in wearable sensors is their use as the sensing layer. For instance, a flexible pH sensor was fabricated with a pH-responsive PANI hydrogel film as the sensing layer.^191^ The device consisted of the PANI sensing layer, a copper interdigital electrode layer, and a polyimide (PI) substrate. It exhibited a sensitivity of 58.57 mV pH^–1^, a low temperature drift, stability under repeated bending, and a response time of under a minute.

### 6.3 CPH in Implantable Sensors

**Figure 8:**
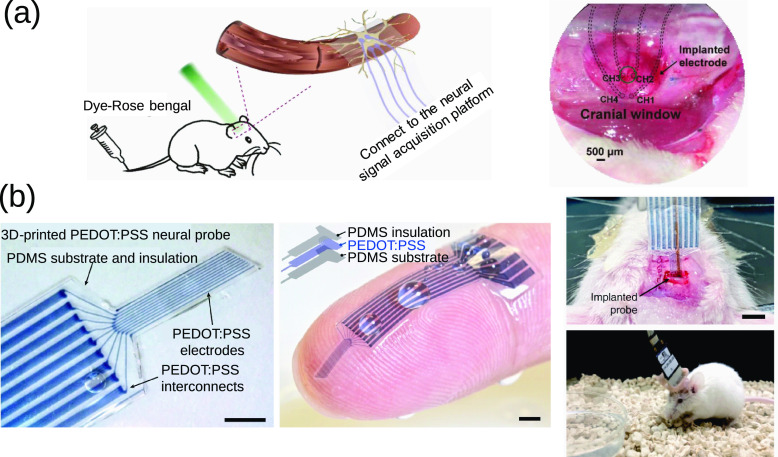
Examples of CPH-based implantable sensors adapted from various sources. (a) Transparent four-channel neural electrode implanted into the primary cortex of a rat. Reproduced from ref (192). Copyright 2021 Wiley. (b) Soft neural probe fabricated from 3D printed PEDOT:PSS on a PDMS substrate and implanted into a freely moving mouse. Reproduced from ref (23). Copyright 2020 Springer Nature Limited.

Although still limited, the use of CPHs in implantable sensors is gaining traction due to their biocompatibility and versatility. One of the main fields of study for this application is in neural electrodes. For example, Zeng et al. proposed the possibility of using PEDOT:PSS hydrogels in neural electrodes.^22^ In this work, the PEDOT:PSS hydrogel was formed via electrogelation on a platinum (Pt) substrate with copper as the sacrificial layer. Compared to bare Pt and PEDOT:PSS-coated Pt, the PEDOT:PSS hydrogel-coated Pt electrode exhibited a lower impedance, a higher charge injection capacity, and a better coating stability after multiple cyclic voltammetry scanning cycles. Although more in vitro and in vivo tests are needed, the study provides a foundation for future work on the use PEDOT:PSS hydrogels in neural electrodes. Likewise, Cui et al. developed a hydrogel-based electrode from a mixture of SF, PEDOT:PSS, and poly(ethylene glycol) diglycidyl ether (PEGDE), which was used as a cross-linker for SF (Figure 8a).^192^ In this work, the authors demonstrated that the addition of PEGDE to SF improved not only the hydrogel’s mechanical properties but also its adhesion to the PEDOT:PSS film layer. The resulting hydrogel exhibited up to a 1000-fold increase in the Young’s modulus and up to a 400% increase in stretchability compared to the SF-only hydrogel. Furthermore, the PEGDE modification of the SF allowed better penetration into the PEDOT:PSS film, hence the improvement in interfacing. Compared to common gold and platinum electrodes, the final hydrogel-based electrode exhibited lower impedance in a biological environment. Moreover, electronic stability was demonstrated for up to four months in PBS. Finally, as a proof of concept, the electrode was implanted in the primary motor cortex of a rat to monitor neural activity during photothrombosis and, in a separate study, in the cortex for anodal stimulation. In both cases, the hydrogel-based electrode showed promising results with low impedance, imaging potential, and a good response time. Similarly, Wang et al. electrodeposited a conductive alginate hydrogel on nickel–cadmium microwires to improve neural electrodes.^193^ In this study, the conductive alginate hydrogel was composed of PEDOT:PSS-coated MWCNT and PEDOT:PSS. The obtained hydrogel-based microwires exhibited good biocompatibility and cell adhesion with SH-SY5Y cells compared to substrates made of ITO, which is typically used as a neural electrode material. Moreover, the hydrogel-coated electrodes gave comparable readings to uncoated electrodes and exhibited good flexibility, which is a desired characteristic of neural electrodes. In another study, a 3D-printable ink made entirely of PEDOT:PSS nanofibrils was fabricated.^23^ In this work, a PEDOT:PSS solution was first submerged in a nitrogen bath, then lyophilized for 72 h in order to obtain the PEDOT:PSS nanofibrils, which were then resuspended in varying concentrations in water and DMSO as the conductive ink (Figure 8b). The resulting conductive ink exhibited a good printing resolution and up to 28 S cm^–1^ of conductivity and 20% maximum strain in the hydrogel state. Moreover, the printed ink in the hydrogel state exhibited constant conductivity of more than 15 S cm^–1^ after 10 000 bending cycles. To demonstrate the use of the conductive ink in biosensors, the ink was printed on PDMS, which served as the substrate and sheathing material, while the conductive ink served as the electrodes of a neural probe. The probe consisted of nine channels, each with a 30 μm feature diameter with an impedance of 50–150 kΩ at 1 kHz. The probe was then implanted into freely moving mice and could successfully record neural activities from each channel over two weeks.

Existing studies demonstrate the potential improvements in tissue interfacing and electrical performances of neural electrodes when incorporated with CPH. However, more extensive in vitro and in vivo long-term studies and analysis of the tissues surrounding the electrode would be of interest to further bolster these early findings.

On the other hand, Ravichandran et al. fabricated an injectable glucose sensor from a double network hydrogel of methacrylated collagen and PPy.^103^ In this system, the hydrogel was cross-linked under physiological conditions and incorporated GOx for the chronoamperometric measurement of glucose. The resulting hydrogel had a conductivity of about 0.0034 S cm^–1^ and a storage modulus of 1 kPa, which could be injected. The PPy in the hydrogel allowed for amperometric measurements of up to 10 mM glucose with repeatable readings with a stability of at least 100 s. The hydrogel was injected in porcine meat, wherein glucose was detected over the course of five days. Although porcine meat cannot represent physiological conditions and further cell culture and in vivo studies must performed to establish performance of the sensor, the work can be considered as an initial proof of concept on the use of CPH-based injectable sensors.

In another study, Kim et al. developed an implantable bladder sensor based on a polypyrrole/agarose hydrogel.^194^ The sensor exhibited a multilevel resistor ladder structure in which the arms started to detach from the structure based on the increase in bladder volume, resulting in an increase in resistance. As a proof of concept, the authors attached the hydrogel sensor ex vivo to a porcine bladder. Although the resolution obtained was quite low, the work demonstrated a possible alternative to current catheter-based bladder volume monitoring devices that exhibited minimal mechanical loading on the bladder and long-term drift.

## 7 Conclusion and Outlook

This Review highlights the works on conducting polymer hydrogels in the field of diagnostic, wearable, and implantable biomedical sensors over the last five years. It also features the properties of the most commonly used CPs and hydrogels and various CPH fabrication techniques. As discussed here, the interest in the use of CPH in biomedical sensors continues to grow, combining CPs with different natural and synthetic hydrogels and reinforcing their electrochemical properties with other CPs or other conductive elements such as MWCNTs and graphene nanosheets.^175,193^ Blending materials takes advantage of the unique features of CPs, resulting in desired sensor properties. In diagnostic biomedical sensors, CPHs are typically used as biocompatible matrices, where catalysts or biorecognition molecules can be well dispersed and target molecules can easily diffuse within, and as conductive matrices capable of rapid charge transfer.^13,14^ Wearable sensors take advantage of the change in CPH resistance during deformation, using CPH mainly in strain and pressure sensors.^17,174^ In addition, CPH has been shown as a possible replacements and/or improvement to ECG and EMG electrodes and wearable diagnostic devices,^189,190^ while in implantable sensors the main application of CPH has been in their use in the improvement of neural electrode interfaces.^22,192^ However, other studies have also shown the possibility of a fully organic implantable sensor based on CPHs.^194^

Despite the significant advances in CPH development, there are still many challenges that need to be addressed prior to their practical application in biomedical sensors. For example, traditional hydrogels lack either stability or sufficient mechanical strength, making them difficult to handle. In addition, traditional hydrogels are difficult to sterilize due to their sensitivity to general sterilization methods, leading to deteriorating sterilization effects.^195^ With regards to CPs, they are still limited by their inferior electroactivity and electrostability compared to metals. In addition, a lot of the work presented in this Review study focused on the investigation of CPHs as suitable materials for biomedical sensors, without an actual functional sensor comprised of the CPH. This is especially true for CPH in wearable pressure and strain sensors. Hence, there is a missing link between the material suitability of CPH and its concrete application in biomedical sensors. Aside from this, in vitro assays conducted are often insufficient to justify moving forward to animal trials. Although animal models suffer from their own issues, such as ethics,^196^ limited animal-to-human translation,^197^ low throughput, and monetary and time expenses,^198^ studying the effect of CPH on multiple organs and on an organism as a whole is crucial especially for CPH in implantable sensors. In relation to this, though CPs are generally considered to be biocompatible,^199^ they are not biodegradable and are combined with other materials to improve their resorbability in vivo.^200^ Furthermore, there is still limited knowledge of the fate of CP monomers and polymers inside the body and the mechanisms of their resorption.

There is still a lot of work to be done on conducting polymer hydrogels to achieve their practical applications in biomedical sensors. The ultimate goal of obtaining completely bioresorbable portable biosensors is still far away, mainly due to the necessary electronics. However, the use of CPH as part of flexible electronic elements is a field that has already begun to be explored. Their unique features and the works presented here show great progress and their potential as candidate materials in the fabrication of all-organic diagnostic, wearable, and implantable sensor devices.

## Abbreviations

AA: acrylic acid
AA-g-NNC: acrylic acid-modified nanocrystal cellulose
ABEI: *N*-(aminobutyl)-*N*-(ethylisoluminol)
APS: ammonium persulfate
ASH: poly(acrylamide-*co*-sodium acrylate)
CME: carbon micromesh electrode
CNT: carbon nanotube
CP: conducting polymer
CPH: conducting polymer hydrogel
DA: dopamine
DBSA: 4-dodecylbenzenesulfonic acid
DMC: methylacryloyl oxygen ethyl trimethylammonium chloride
DMSO: dimethyl sulfoxide
ECL: electrochemiluminescent
EG: ethylene glycol
EIS: electrochemical impedance spectroscopy
ELISA: enzyme-linked immunosorbent assay
GCE: glassy carbon electrode
GDP: gross domestic product
GO: graphene oxide
GOx: glucose oxidase
HEAA: *N*-hydroxyethyl acrylamide
HMEDOT: hydroxymethyl (3,4-ethylenedioxythiophene)
HOMO: highest occupied molecular orbital
LUMO: lowest unoccupied molecular orbital
IPN: interpenetrating network
LCST: lower critical solution temperature
LOD: limit of detection
MAA: methacrylic acid
MMA: methyl methacrylate
MWCNT: multiwall carbon nanotube
NIR: near-infrared
NP: nanoparticle
L-Dopa: levodopa
PA: phytic acid
PAA: poly(acrylic acid)
PANI: polyaniline
PAM: polyacrylamide
PCL: polycaprolactone
PDA: polydopamine
PDEAAM: poly(*N*,*N*-diethylacrylamide)
PDMS: polydimethylsiloxane
PEDOT: poly(3,4-ethylenedioxythiophene)
PEGDA: poly(ethylene glycol diacrylate)
PEGDE: poly(ethylene glycol) diglycidyl ether
PEO: poly(ethylene oxide)
PGS: poly(glycerol sebacate)
pHEMA: poly(hydroxyethylmethacrylate)
PHMEDOT: poly hydroxymethyl (3,4-ethylenedioxythiophene)
PLA: polylactic acid
PSS: poly(4-styrenesulfonate)
PLGA: poly(lactic-*co*-glycolic acid)
PEG: poly(ethylene glycol)
PMMA: poly(methacrylic acid)
PEGMEA: poly(ethylene glycol) methyl ether acrylate
γPGA: poly-γ-glutamic acid
PNIPAm: poly(*N*-isopropylacrylamide)
PPy: polypyrrole
PU: polyurethane
PVA: poly(vinyl alcohol)
PVP: poly(vinylpyrrolidone)
QD: quantum dot
rGO: reduced graphene oxide
SBAA: *N*-(3-sulfopropyl)-*N*-methacryloylamidopropyl-*N*,*N*-dimethylammonium betaine
SEM: scanning electron microscopy
SIPN: semi-interpenetrating polymer network
SF: silk fibroin
SS: silk sericin
TA: tannic acid
TENG: triboelectric nanogenerator
TOCNF: TEMPO-oxidizedcellulose nanofiber
UA: uric acid
UCST: upper critical solution temperature
ZFS: Zonyl FS-300.
